# Functionalization of Graphene Oxide with Polysilicone: Synthesis, Characterization, and Its Flame Retardancy in Epoxy Resin

**DOI:** 10.3390/polym13213857

**Published:** 2021-11-08

**Authors:** Jiangbo Wang

**Affiliations:** School of Materials and Chemical Engineering, Ningbo University of Technology, Ningbo 315211, China; jiangbowang@nbut.edu.cn

**Keywords:** graphene oxide, polysilicone, functionalization, flame retardancy, epoxy resin

## Abstract

A novel polysilicone flame retardant (PMDA) has been synthesized and covalently grafted onto the surfaces of graphene oxide (GO) to obtain GO-PMDA. The chemical structure and morphology of GO-PMDA was characterized and confirmed by the Fourier transform infrared (FTIR) spectroscopy, X-ray photoelectron spectrometer (XPS), atomic force microscope (AFM), and thermogravimetric analysis (TGA). The results of dynamic mechanical analysis (DMA) indicated that the grafting of PMDA improved the dispersion and solubility of GO sheets in the epoxy resin (EP) matrix. The TGA and cone calorimeter measurements showed that compared with the GO, GO-PMDA could significantly improve the thermal stability and flame retardancy of EP. In comparison to pure EP, the peak heat release rate (pHRR) and total heat release (THR) of EP/GO-PMDA were reduced by 30.5% and 10.0% respectively. This greatly enhanced the flame retardancy of EP which was mainly attributed to the synergistic effect of GO-PMDA. Polysilicone can create a stable silica layer on the char surface of EP, which reinforces the barrier effect of graphene.

## 1. Introduction

Epoxy resin (EP) was widely used in various industrial fields such as coating, adhesives, laminates, and composites, etc., [1,2,3,4,5]. However, one of the main drawbacks of epoxy resin is its inherent flammability, which restricts its application in many fields for safety consideration. Therefore, it is an important issue to improve the flame retardancy of EP. Halogen-free flame retardants (such as hydrated alumina, aromatic phosphates, etc.) with their environment friendly property has become a new trend of replacing the original position of halogen-containing flame retardants in improving fire resistance of epoxy resin [6,7,8].

As the two-dimensional sp2-hybridized carbon, graphene is currently the most intensively studied material. This single-atom-thick sheet of carbon atoms arrayed in a honeycomb pattern is the world’s thinnest, strongest, and stiffest material [9,10]. Until recently, most studies of graphene-based nanocomposites focused on the incorporation of graphene, modified graphene, or graphene oxide (GO) into polymer matrices. Because of their layered structures, graphene and GO can act as barriers reducing the heat released and insulating against the transfer of combustion gases into the inflammable polymer matrix [11,12,13,14,15].

However, due to the high surface area and strong Van der Waals force, the re-aggregating phenomenon is inclined to appear between graphene sheets, which limit its use in polymer matrix [16,17]. The problem is usually solved by covalent functionalization. After oxidation, rich oxygen-containing groups (e.g., hydroxyl, epoxide, carboxyl and carbonyl groups, etc.) are brought to the surface of graphene sheets. Through further chemically functionalizing the GO, grafting the organic molecules on GO was widely adopted in improving the dispersion and thermal stability of GO [18,19,20,21]. Polyhedral oligomeric silsesquioxane (POSS, which contains an inner inorganic framework made up of silicone and oxygen (SiO_1.5_)_x_ and is externally covered by organic substituents.), phosphorus-containing molecule, and intumescent flame retardant have been designed and covalently grafted onto the surface of graphene sheets to obtain a novel flame retardant [22,23,24,25,26,27]. In our previous research [28,29], polysiloxane was grafted onto the surface of carbon nanotubes, which not only improved the dispersion of carbon nanotubes in matrix, but also improved the flame retardancy of the epoxy resin and thiol-ene polymer.

In this study, a novel polysilicone (PMDA), which consisted of 60 mol% phenylsiloxane, 35 mol% methylsiloxane, and 5 mol% aminosiloxane as a unit, was synthesized by the hydrolysis and polycondensation method. Subsequently, PMDA was grafted onto the surface of graphene oxide to improve the dispersion and flame retardancy of graphene in the polymer. The results of Fourier transform infrared (FTIR) spectroscopy, X-ray photoelectron spectrometer (XPS), atomic force microscope (AFM), and thermogravimetric analysis (TGA) measurements indicated that the polysilicone has been successfully attached to the surfaces of GO. It is confirmed that the covalent grafting of polysilicone onto graphene sheets improved both the flame retardancy efficiency and the dispersibility of graphene in the EP matrix, which provides a new path to obtain a new kind of graphene- based flame-retardant nanocomposites.

## 2. Materials and Methods

### 2.1. Materials

Graphite powders (spectrum pure), concentrated sulfuric acid (98%), phosphoric acid (85%), potassium permanganate, hydrogen peroxide (30%), tetramethylammonium hydroxide (TMAOH), methyltrimethoxysilane (MTMS), (3-aminopropyl)trimethoxysilane (APT), *N,N′*-dicyclohexylcarbodiimide (DCC) and tetrahydrofuran (THF) were all purchased from Alfa Aesar Chemical Reagent Co., Ltd. (Tewksbury, MA, USA). Phenyltrimethoxysilane (PTMS) and dimethyldimethoxysilane (DMDS) were reagent grade and purchased from Gelest Chemical Reagent Co., Ltd. (Morrisville, PA, USA). Ethyl alcohol was supplied by Sigma-Aldrich Reagent Co., Ltd. (St. Louis, MO, USA). Chloroform (CHCl_3_) and hydrochloric acid were supplied by Fisher Scientific Chemical Co. (Waltham, MA, USA). EPON 826 with an epoxy equivalent weight of 178–186 g was supplied by Hexion Specialty Chemicals Inc. (Columbus, OH, USA) and used as received. The hardener, Jeffamine D230, with an amine equivalent weight of 60 g, was supplied by Huntsman Corp. (Woodlands, TX, USA) and also used as received.

### 2.2. Synthesis of Polysilicone (PMDA)

**Scheme 1:**
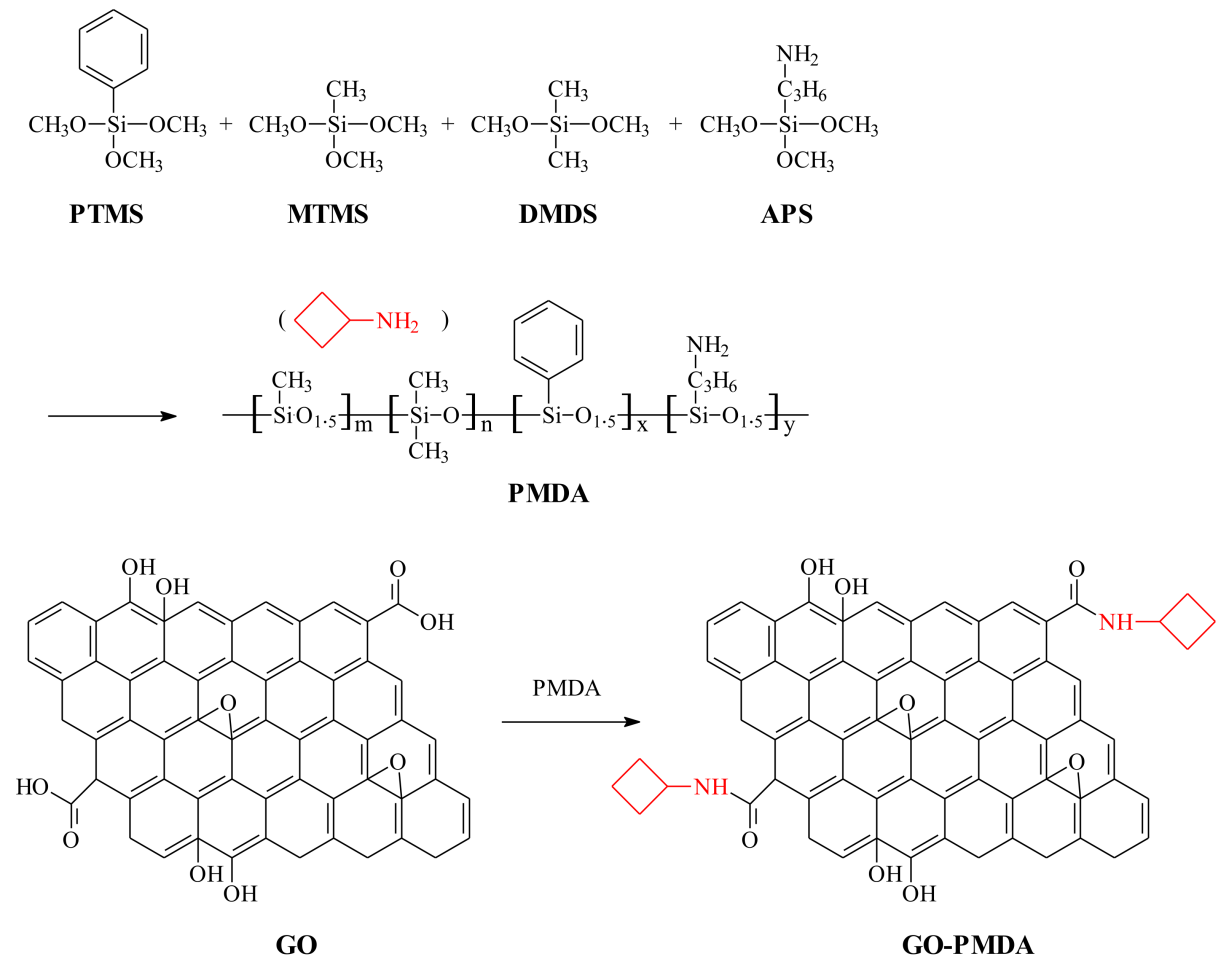
Synthesis route of GO-PMDA.

The polysilicone PMDA was synthesized by the hydrolysis and polycondensation method as shown in Scheme 1. The raw material consisted of 60 mol% phenylsiloxane, 35 mol% methylsiloxane and 5 mol% aminosiloxane as a unit. The ratio of organic groups to silicon atoms (R/Si) was 1.2, which was used to characterize the extent of branches to a polysiloxane structure; the molecular structure of polysilicone is illustrated in Scheme 1. Distilled water (25 mL), EtOH (75 mL), and TMAOH (1 mL) were mixed in a 250 mL flask under stirring, followed by adding the mixture of PTMS, MTMS, DMDS, and APS at certainly molar ratios (0.69:0.06:0.20:0.05) and maintaining 10% weight percentage solution. The stirring was maintained for 8 h, and the resulting solution was stored at room temperature overnight. Through decantation of most clear supernatant, precipitated condensate was collected and then washed by vacuum filtration with distilled H_2_O/EtOH (1/3 by volume), then washed again in pure EtOH. Finally, the acquired rinsed powder (PMDA) was thoroughly dried under vacuum for 20 h at room temperature [28].

### 2.3. Functionalization of Graphene Oxide (GO)

GO was prepared from graphite by modified Hummers’ method [30]. In a 500 mL three-neck flask, the as-prepared GO (0.2 g) was first suspended in THF (200 mL) under ultrasonication for 90 min. Subsequently, the PMDA (0.8 g) and DCC (0.1 g, as cat.) were introduced into the above flask, and followed by ultrasonication for 30 min. With stirring, the mixture was heated to 66 °C and refluxed for 20 h under nitrogen atmosphere. Afterwards, the mixture was centrifuged and thoroughly washed with anhydrous THF to remove the residual PMDA. Then, the product dried in a vacuum at room temperature for 12 h to remove the solvent (Scheme 1).

### 2.4. Preparation of Epoxy Composite

Briefly, the EP/GO-PMDA composites were prepared as follows: The GO-PMDA (2 g) was dispersed in acetone and sonicated for 60 min to form a uniform black suspension. Then, EPON 826 (73.5 g) was added to the mixture and dispersed by a mechanical stirrer for 30 min. The mixture was heated in a vacuum oven at 50 °C for 10 h to remove the solvent. After that, D230 (24.5 g) was added into the mixture and stirring for 30 min. After degassing in vacuum for 10 min to remove any trapped air, the samples were cured at 80 °C for 2 h and post cured at 135 °C for 2 h. For comparison, pure epoxy (EP) and 2 wt% GO/epoxy (EP/GO) composites were also prepared at same processing condition.

### 2.5. Characterization and Measurement

The Fourier transform infrared spectroscopy (FTIR) was tested using a Digilab Scimitar FTS-2000 IR spectrometer (Digilab Inc., Hopkinton, MA, USA) at a resolution of 2 cm^−1^ with 20 scans. The samples were mixed with potassium bromide and pressed to a disc, which was used for measurement. X-ray photoelectron spectroscopy (XPS) was carried out in a Thermo Scientific ESCALAB 250Xi X-ray photoelectron spectrometer (Thermo Fisher Scientific Inc., Waltham, MA, USA) equipped with a mono-chromatic Al Kα X-ray source (1486.6 eV). AFM observation was performed on the Bruker Dimension Icon atomic force microscope (Bruker Corp., Karlsruhe, Germany) in tapping-mode. The aqueous GO suspension and DMF suspension of GO-PMDA were spin-coated onto freshly cleaved silica surfaces. Thermogravimetric analysis (TGA) was carried out on a TA instrument Q5000 thermogravimetric analyzer (TA Instrument Corp., New Castle, DE, USA). The sample was dried in an oven at 100 °C for 5 h to remove moisture, and then about 10 mg sample was heated from 50 to 700 °C at a 10 °C/min heating ramp rate in nitrogen atmosphere. Dynamic mechanical analysis (DMA) was determined using a Rheometric Scientific SR-5000 dynamic mechanical analyzer (Rheometric Scientific Inc., West Yorkshire, UK). Data were collected from 60 to 120 °C at a scanning rate of 5 °C/min. Cone calorimeter measurement was performed on an FTT cone calorimeter (Fire Testing Technology Ltd., East Grinstead, West Sussex, UK) according to ASTM E1354. The dimension of each specimen was 100 × 100 × 3 mm^3^. All the measurements were repeated three times and the results were averaged.

## 3. Results and Discussion

### 3.1. Structural Characterization

**Figure 1:**
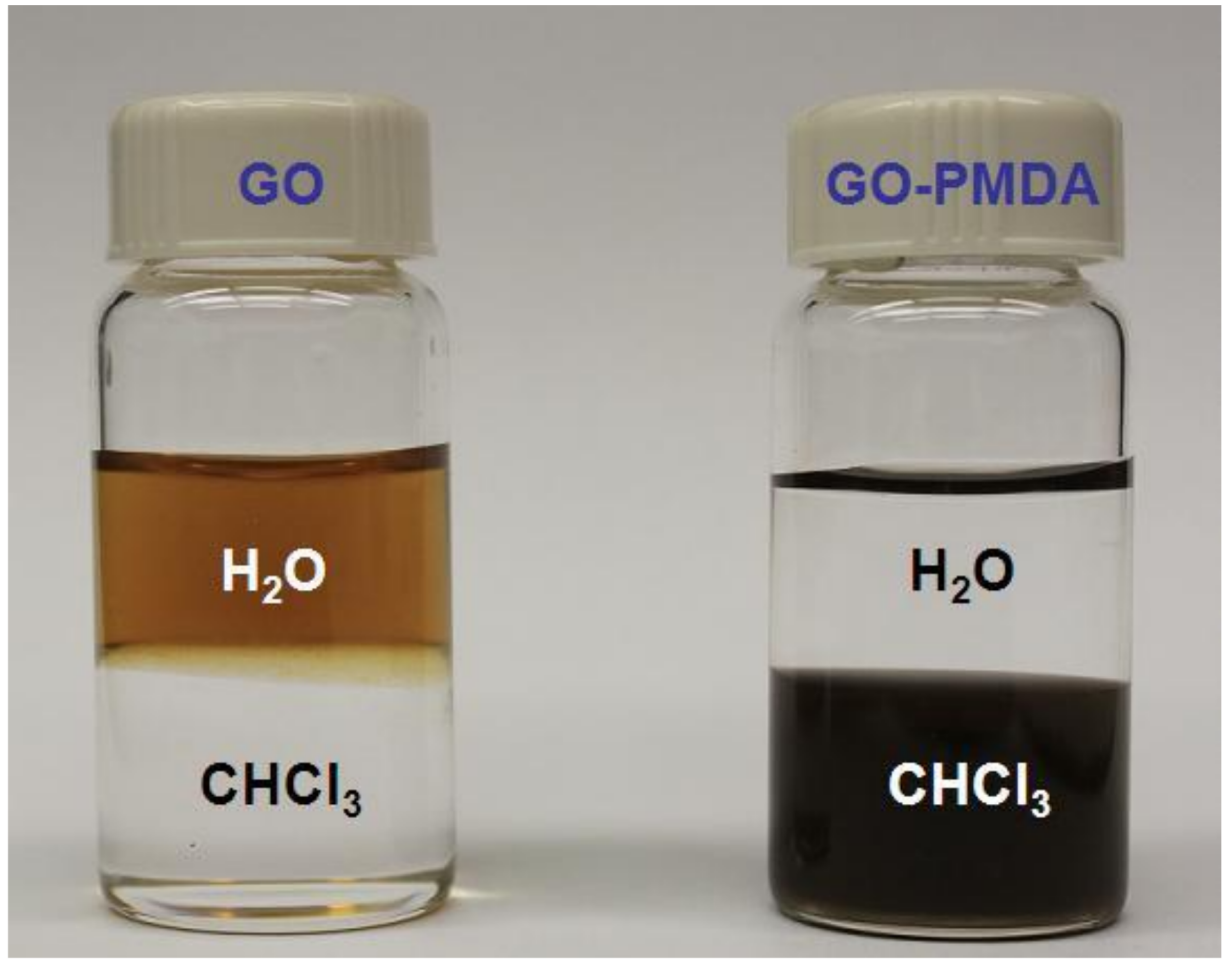
Solubility of GO and GO-PMDA.

The dispersion of GO-PMDA is very important for the preparation of GO-PMDA polymer nanocomposites. Figure 1 shows the digital photos of dispersion stability of low-concentration GO and GO-PMDA (0.5 mg/mL) in insoluble mixtures of water and CHCl_3_ after 24 h of static placement.

As is well-known, carboxylic, epoxy, carbonyl, and hydroxide groups are present on the surface and edge of GO sheets, which make GO hydrophilic. Therefore, it can be found that the GO homogeneously disperses in water and exhibits a light yellow color. Meanwhile, the stability of the dispersion of GO-PMDA is very good in CHCl_3_ without noticeable precipitation. The strong interaction between PMDA and CHCl_3_ makes the dispersion of GO-PMDA in CHCl_3_ stable. The evolution of surface functionality during reaction usually leads to a change of the graphene sheet from hydrophilic into hydrophobic. Moreover, the color of GO gradually changes to black after the one-step functionalization and reduction process, which is usually considered to be a sign of GO reduction.

**Figure 2:**
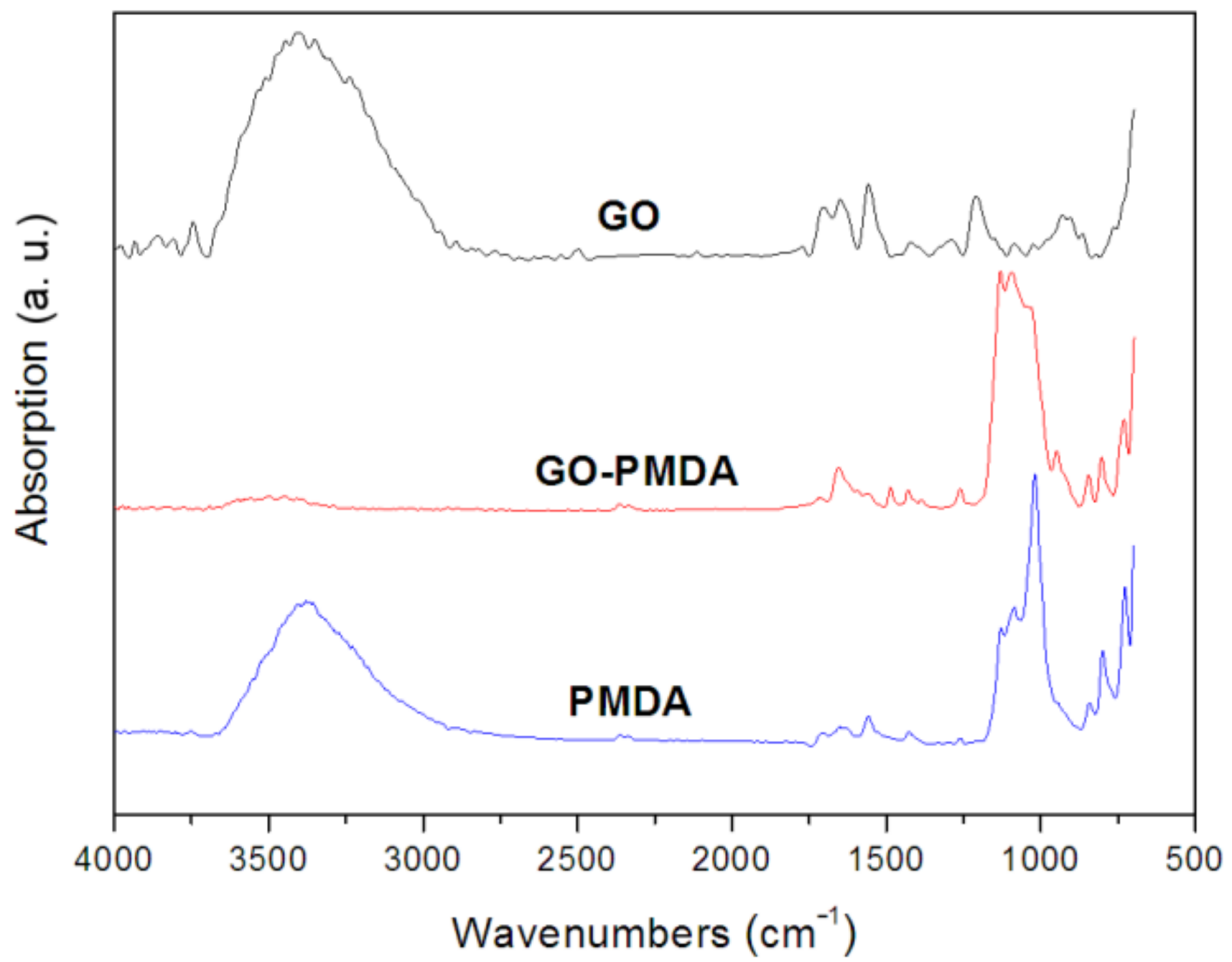
FTIR spectra of GO, PMDA, and GO-PMDA.

FTIR spectra for the GO, PMDA, and GO-PMDA is measured in Figure 2. As expected, GO presents the typical spectra of oxygen-based functional groups: –OH at 3408 cm^−1^, C=O at 1705 cm^−1^, C–O at 1211 cm^−1^. The intensities of these IR peaks decrease significantly after chemical attachment of PMDA onto the GO. In addition, the strong peak at 1200–1000 cm^−1^ characteristic of the Si-O-Si stretching vibration in PMDA is clearly seen in the FTIR spectra of GO-PMDA, indicating that PMDA has been successfully grafted onto GO.

**Figure 3:**
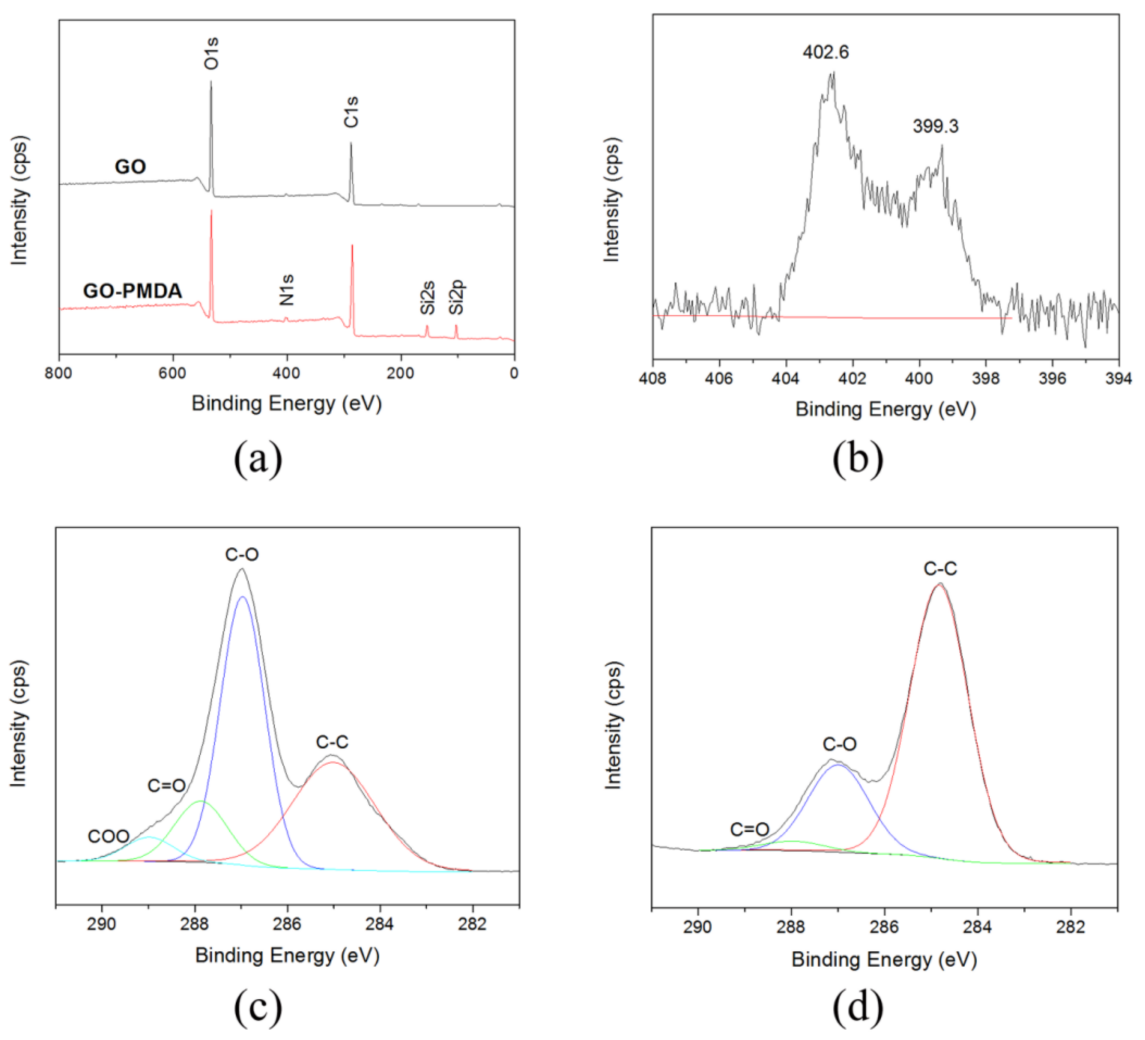
XPS survey spectra of the GO and GO-PMDA (**a**) and high-resolution XPS spectra of N1s for GO-PMDA (**b**), C1s for GO (**c**), C1s for GO-PMDA (**d**).

XPS measurement is performed to gain more information about chemical bonds formed on the surface of GO before and after its functionalization with PMDA. As expected, only C1s and O1s peaks are obtained in the XPS survey spectra for GO (Figure 3a). Compared to that of GO, the XPS survey spectra for GO-PMDA shows additional N1s, Si2s, and Si2p peaks, accompanied by the reduced O1s peak with respect to the C1s peak. Furthermore, it is easy to see the N1s band of GO-PMDA locates at 399.3 eV (C–N) and 402.6 eV (CO–N) in Figure 3b, which further proves that GO is modified with PMDA through the success of the covalent bonding of PMDA onto GO.

—COOH + NH_2_— → —CO—NH— (1)

—CH(O)CH_2_ + NH_2_— → —CHOHCH_2_—NH— (2)

Figure 3c,d reproduces the high-resolution C1s spectra for GO and GO-PMDA, respectively. The peaks for C–C (285.0 eV), C–O (287.0 eV), C=O (287.9 eV), and COO (289.0 eV) are clearly observed in C1s scan of GO (Figure 3c). As can be seen, the COO peak at 289.0 eV for GO-PMDA almost disappears upon the amide formation with the amine group of PMDA. The peak intensity of the C–O (287.0 eV) and C=O (287.9 eV) in GO-PMDA also significantly decreases (Figure 3d). This is because the nucleophilic substitution between GO and amine groups can cause deoxygenation and reduction of graphene oxide [27].

**Figure 4:**
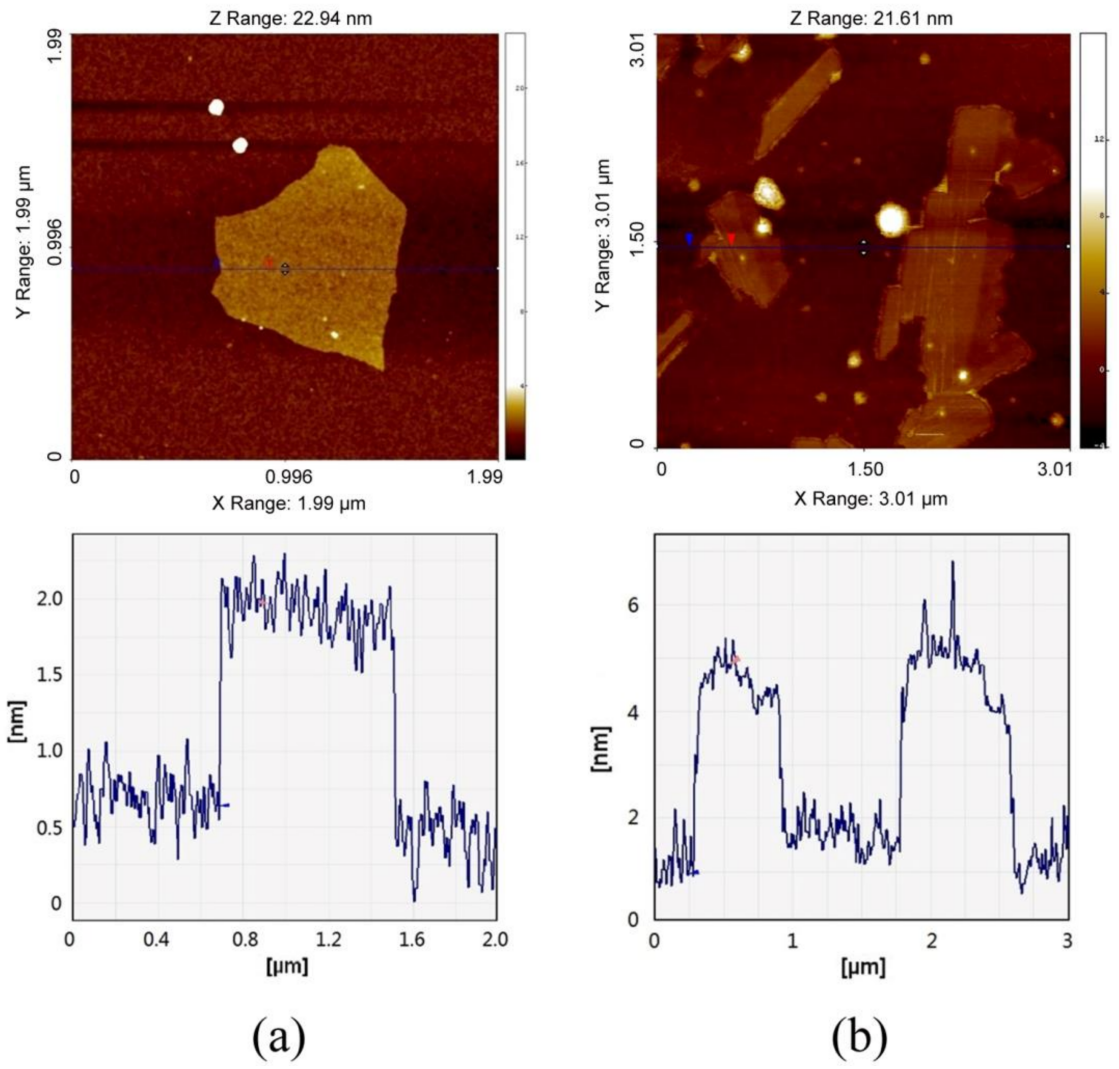
AFM images and corresponding height profiles of GO (**a**), GO-PMDA (**b**).

AFM measurement is performed to investigate the morphology and thickness of the interface layer grafted on the GO sheet surface. Figure 4a represents the tapping mode AFM images of GO, showing the average height of ~1 nm for a single-layer GO sheet. After grafting PMDA, the height of a single-layer GO-PMDA becomes ~4 nm (Figure 4b), which is much higher than that of GO. The thicker sheet is possibly due to the PMDA chain grafted on GO sheet surface which indicates that the GO-PMDA is successfully obtained in our work.

**Figure 5:**
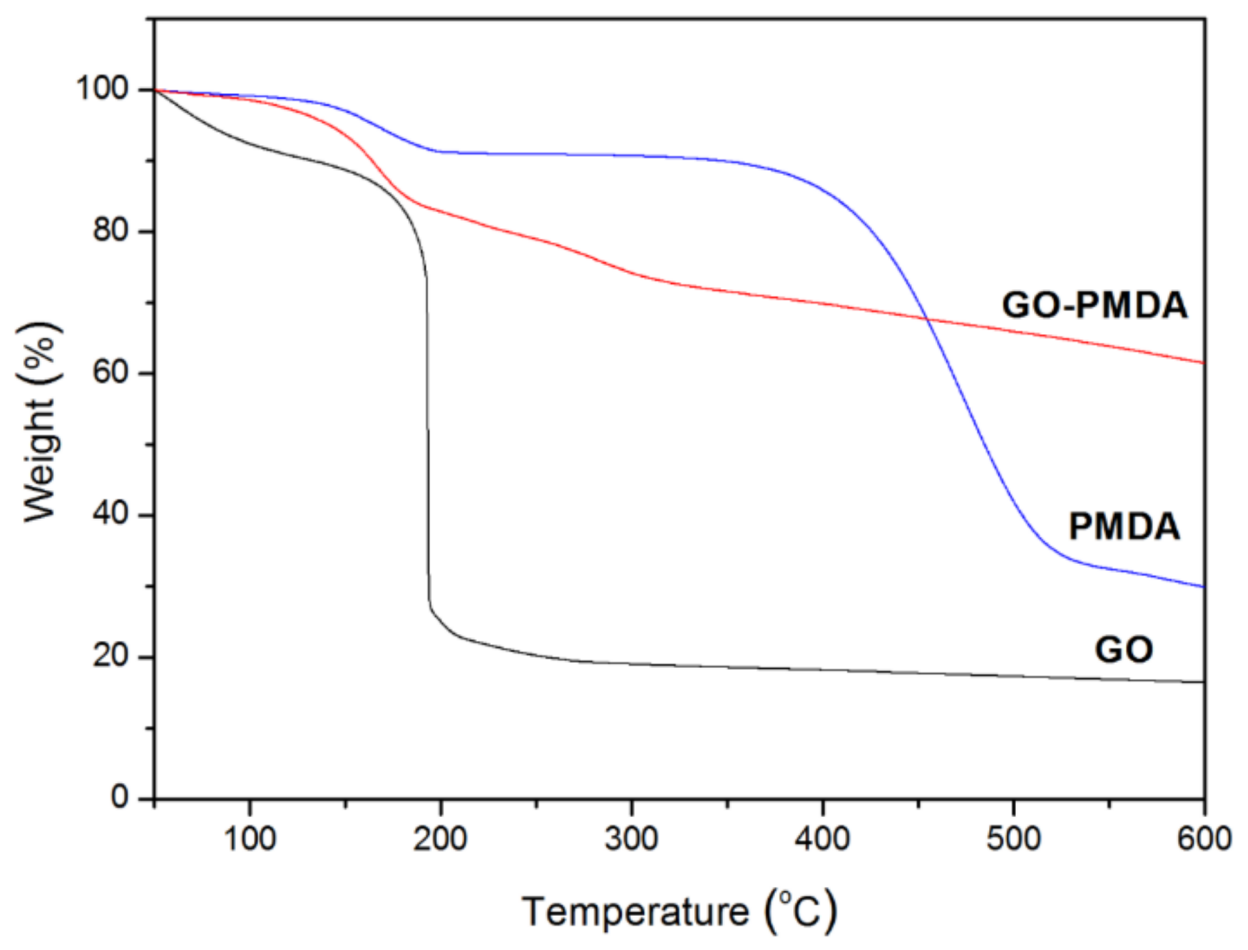
TGA curves of GO, PMDA, and GO-PMDA.

TGA curves of GO, PMDA, and GO-PMDA are shown in Figure 5. The initial 7.6 wt% weight loss seen for GO up to 100 °C is associated with the thermal desorption of water molecules physically adsorbed onto the hydrophilic GO surface. The main weight loss of GO is found around 190–200 °C because of the decomposition of oxygen-containing functional groups to CO, CO_2_, and H_2_O [27]. The weight loss between 200 °C and 600 °C is about 8.5 wt%, associated with the removal of more thermally stable oxygen functionalities and thermal decomposition of GO. Compared with the onset degradation temperatures (*T*_5wt%_, 78.5 °C) and the char yield (16.5 wt% at 600 °C) of GO, the TGA curve of GO-PMDA exhibits a much better thermal stability with both higher of *T*_5wt%_ (141.9 °C) and char yield (61.5 wt% at 600 °C). These results can be attributed to the grafting of PMDA, which is a good char layer stabilizer. Therefore, the char-forming performance of graphene can be greatly improved, so as to better play the role of heat insulation and isolation of combustible substances.

### 3.2. Dispersion

**Figure 6:**
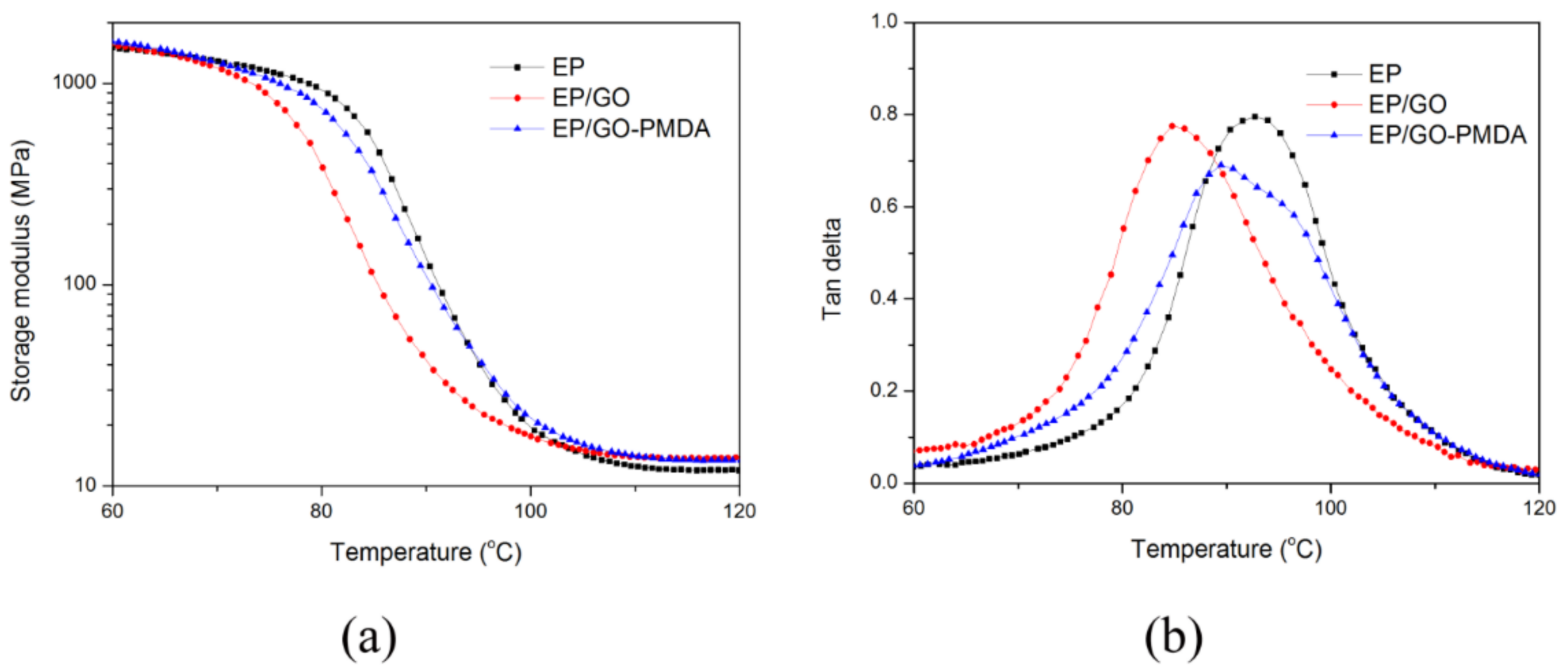
Storage modulus (**a**) and Tan delta (**b**) curves for EP, EP/GO, and EP/GO-PMDA.

DMA test is always used to evaluate the interfacial interaction between additives and the polymer matrix. Figure 6 shows the temperature dependence of the storage modulus and tan delta of EP and its nanocomposites. The storage modulus of pure EP in Figure 6a at 80 °C is 928.2 MPa. When adding 2 wt% of GO, the storage modulus of EP composites is decreased by 57.7% and reaches 392.9 MPa below the *T_g_*. The high hydrophilic of GO and its large aspect ratio have a significant effect on the decrease of storage modulus. This is ascribed to that GO affects the cross-linked structure between the EP molecular chains and the curing agent, and a plasticizer role played by GO increases the flexibility of chain segments of EP matrix, which means the reduced cross-linking density of EP will lead to decreased mechanical properties [31]. However, the storage modulus of EP/GO-PMDA shows much higher increase compared with EP/GO. The same tendency is found in *T_g_* values. The addition of 2 wt% GO decreases *T_g_* of the EP matrix from 92.7 °C to 84.8 °C, compared with which, the addition of GO-PMDA increases *T_g_* of EP/GO from 84.8 °C to 89.5 °C. This indicates that the grafting of PMDA improves the dispersion and solubility of GO sheets in the EP matrix.

### 3.3. Thermal Stability

**Figure 7:**
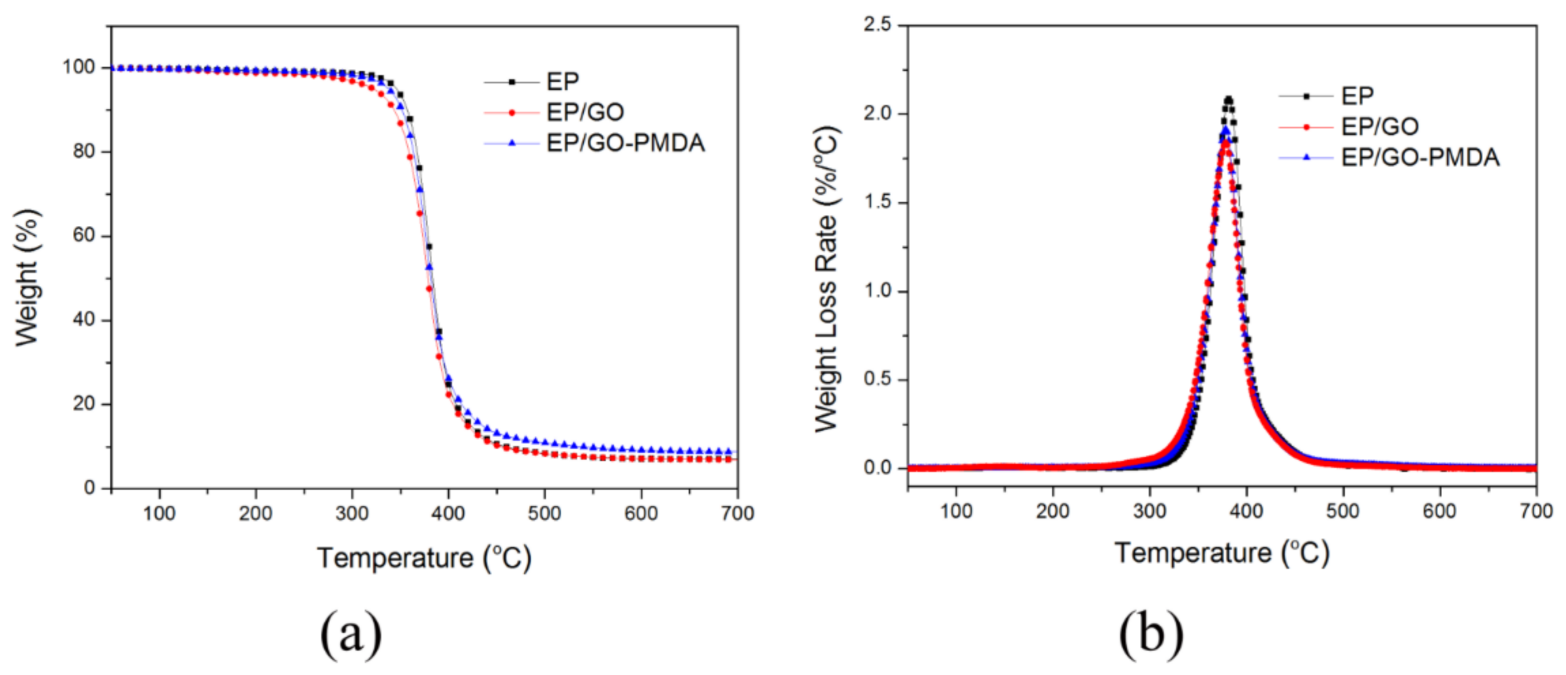
TGA (**a**) and DTG (**b**) curves of EP, EP/GO, and EP/GO-PMDA.

The thermal stability of EP, EP/GO, and EP/GO-PMDA under nitrogen atmosphere is measured by TGA (Figure 7). Related data are listed in Table 1. The *T*_5wt%_ of EP is 346 °C, compared with which, the *T*_5wt%_ of EP/GO is lower than pure EP, since GO is thermally unstable and its major weight loss occurs below 200 °C due to the decomposition of the oxygen-contained functional moieties. The addition of GO-PMDA exhibits a same trend in the *T*_5wt%_ compared with GO. Even though, the *T*_5wt%_ of EP/GO-PMDA is increased by 15.4 °C compared with that of EP/GO. The *T*_max_ of the EP/GO and EP/GO-PMDA is quite similar to EP, however, DTG peak rates of the EP/GO and EP/GO-PMDA, which indicate thermal degradation rates, are visibly decreased. Furthermore, the residual char obtained from EP/GO-PMDA is remarkably higher than EP and EP/GO, increased by 1.6 wt% with only 2 wt% addition. The rich char yield formed during decomposition is due to the condensed phase flame retardant mechanism of silicon element in GO-PMDA, which can block the fuel and oxygen between composites and the environment as well as hinder the heat transfer.

### 3.4. Flame Retardancy

**Figure 8:**
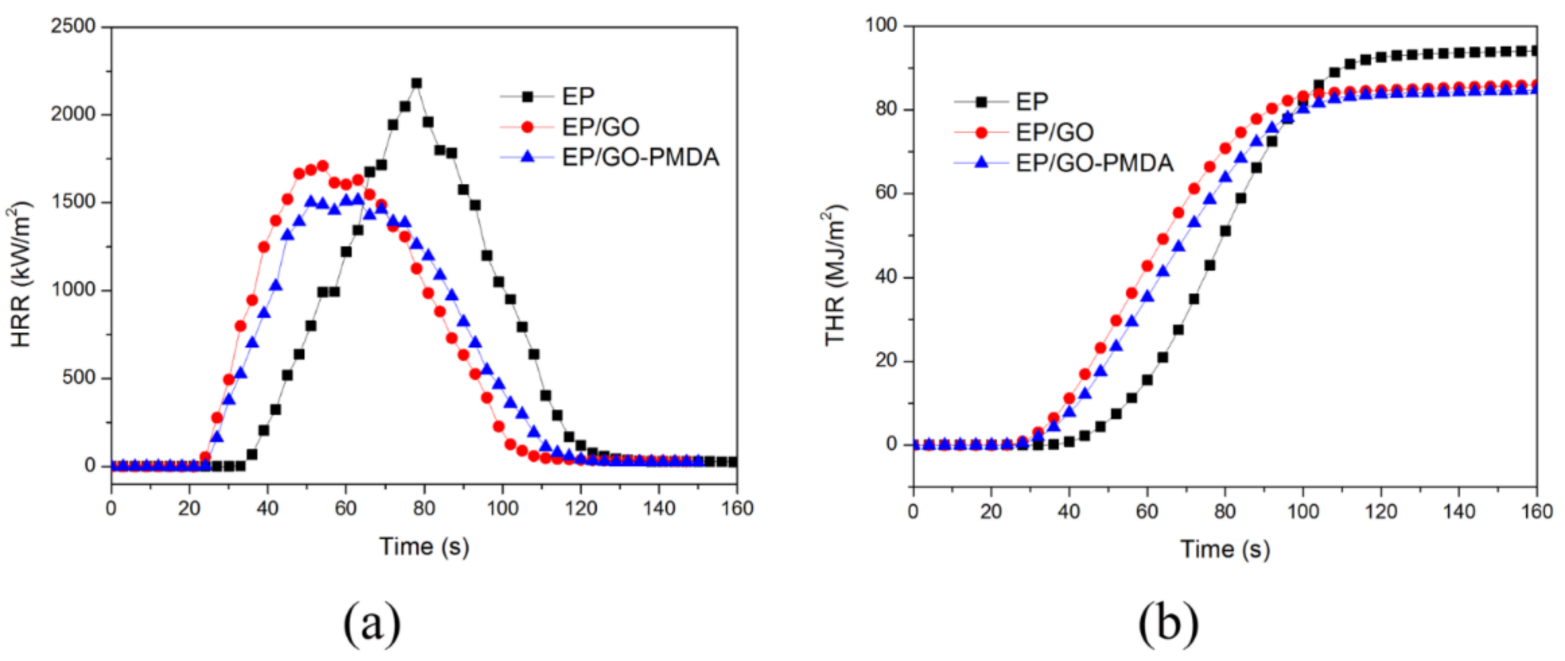
HRR (**a**) and THR (**b**) curves of EP, EP/GO, and EP/GO-PMDA.

The cone calorimeter is one of the most effective methods to evaluate the flammability of various composites in real-world fire conditions. The heat release rate (HRR) and total heat release (THR) obtained from cone calorimeter have been found to be important parameters to evaluate fire safety. Figure 8 shows the HRR and THR versus time curves of EP, EP/GO, and EP/GO-PMDA. In comparison to pure EP, the peak heat release rate (pHRR) and THR of nanocomposites with the incorporation of 2 wt% GO was reduced by 21.6% and 8.8% respectively. The superior flame retardancy of EP/GO over EP could be attributed to the barrier effect of GO, which retards the permeation of heat and the escape of volatile degradation products. Moreover, the pHRR of EP/GO-PMDA in Figure 8a exhibits further reduction (30.5%, compared with EP), even though THR in Figure 8b displays little change (10.0%, compared with EP). The best flame-retardant properties of EP/GO-PMDA could be attributed to two aspects: first, the reduction of GO by PMDA occurs to convert GO into a more stable form, reduced-GO; second, PMDA can create a stable silica layer on the char surface of EP, which reinforces the barrier effect of graphene, simultaneous reduction and surface functionalization of graphene oxide with PMDA for reducing fire hazards in epoxy composites.

## 4. Conclusions

In this paper, PMDA has been covalently grafted onto the surfaces of GO to prepare GO-PMDA. The results from FTIR, XPS, AFM, and TGA measurements showed that GO-PMDA was successfully grafted onto the surface of GO. The functionalization of GO with PMDA made the hydrophilic GO hydrophobic. DMA test exhibited that the grafting of PMDA improved the dispersion of GO sheets in EP matrix. Furthermore, GO-PMDA could significantly improve thermal stability and flame retardancy of EP compared with the GO. The peak heat release rate (pHRR) and total heat release (THR) of EP/GO-PMDA were reduced respectively. This greatly enhanced flame retardancy of EP by GO-PMDA which was mainly attributed to the synergistic effect of polysilicone and graphene. The PMDA grafting method used in this study can simultaneously improve the dispersion and flame retardancy of graphene in epoxy materials. This will help to further promote the application process of graphene and develop high-performance polymer materials.

## Figures and Tables

**Table 1:** TGA data of EP, EP/GO, and EP/GO-PMDA.

Sample	Temperature (°C)			Peak Rate (wt%/°C)	Residues (wt%)
	*T*_5wt%_	*T*_50wt%_	*T*_max_		
EP	346.0	383.5	381.4	2.09	7.02
EP/GO	322.5	378.6	378.2	1.84	6.95
EP/GO-PMDA	337.9	381.3	378.2	1.92	8.76

## Data Availability

The data used to support the findings of this study are available from the corresponding author upon request.
